# Influence of Bond Coat Roughness on the Microstructure and Mechanical Properties of EB-PVD TBCs

**DOI:** 10.3390/ma19183857

**Published:** 2026-09-10

**Authors:** Grzegorz Maciaszek, Andrzej Nowotnik, Julia Maciaszek

**Affiliations:** Faculty of Mechanical Engineering and Aeronautics, Rzeszów University of Technology, 35-959 Rzeszów, Poland; g.maciaszek@prz.edu.pl (G.M.);

**Keywords:** thermal barrier coatings, TBCs, electron-beam physical vapour deposition, EB-PVD, indentation, coating hardness

## Abstract

The surface condition of the bond coat is an important factor governing the growth and microstructural development of electron-beam physical vapour deposition (EB-PVD) TBCs. In this study, 7 wt.% yttria-stabilised zirconia (7YSZ) coatings were deposited by EB-PVD on vapour-phase aluminide bond coats with three distinct surface conditions, ranging from the as-coated state to ground and polished surfaces. The effect of bond coat roughness was evaluated through surface profilometry, scanning electron microscopy, X-ray diffraction, quantitative analysis of columnar architecture and porosity, scratch testing, and Vickers microindentation. Reducing the bond coat roughness resulted in a more uniform columnar architecture, reduced intercolumnar spacing, and a lower degree of column inclination, accompanied by a decrease in the coating surface roughness. XRD confirmed the formation of the expected tetragonal/cubic 7YSZ phase assemblage for all investigated conditions. Scratch testing revealed no delamination or spallation up to the maximum applied load of 200 N, indicating high interfacial integrity irrespective of bond coat roughness. Vickers measurements revealed a statistically significant through-thickness hardness gradient for all coatings, with hardness decreasing from the bond coat interface towards the column tips. Importantly, bond coat roughness had no statistically significant effect on hardness near the interface or in the central region, whereas significant differences were observed near the column tips. These results demonstrate that bond coat roughness strongly governs EB-PVD columnar architecture and surface morphology, while its influence on hardness is predominantly confined to the uppermost region of the coating.

## 1. Introduction

Thermal barrier coatings (TBCs) are widely used to protect components operating under severe thermal and environmental conditions in gas turbines and aero-engine systems, particularly in high-pressure turbine sections [1]. Their primary function is to reduce the temperature of metallic components exposed to hot combustion gases, thereby mitigating high-temperature degradation mechanisms [2]. The application of TBC systems enables an increase in the turbine inlet temperature, which directly contributes to improved engine efficiency and reduced fuel consumption [3,4].

Conventional TBC systems consist of a multilayer architecture comprising a metallic bond coat and a ceramic top coat [5]. The bond coat, typically based on MCrAlY alloys (M = Ni and/or Co) or diffusion aluminides, is deposited directly onto the substrate and serves multiple functions, including oxidation protection, mitigation of thermally induced stresses, and promotion of adhesion between the substrate and the ceramic layer [6]. The ceramic top coat, most commonly yttria-stabilised zirconia (YSZ), provides thermal insulation and protects the underlying metallic components from exposure to the hot gas stream [7].

Among the available deposition techniques, electron-beam physical vapor deposition (EB-PVD) is widely employed for the production of advanced TBC systems because of its ability to generate a strain-tolerant columnar microstructure [8]. During deposition, ceramic columns with low defect density and controlled intercolumnar porosity grow preferentially perpendicular to the substrate surface [9,10]. This characteristic architecture enhances the strain accommodation capability of the coating and improves its resistance to thermomechanical fatigue and thermal shock. Furthermore, EB-PVD coatings exhibit relatively low surface roughness, good adhesion to the substrate, and enhanced resistance to erosion, making them particularly suitable for aero-engine applications [11].

The microstructure and performance of the EB-PVD coatings are strongly influenced by both the processing conditions and the surface characteristics of the bond coat prior to deposition [12,13]. Important process parameters include substrate temperature, chamber pressure, substrate rotation, and vapour flux conditions [14]. In addition to these variables, the surface topography of the bond coat constitutes an important initial condition for ceramic nucleation and subsequent column growth [15]. Variations in surface roughness and local surface inclination can modify the distribution of nucleation sites and the local growth conditions, thereby affecting column orientation, morphology, density, and intercolumnar spacing. Previous studies [16] have demonstrated that changes in bond coat roughness can influence the crystallographic texture and microstructural development of EB-PVD YSZ coatings, as reflected, for example, in variations in the relative intensity of characteristic X-ray diffraction reflections [17].

The influence of bond coat topography may extend beyond the initial nucleation stage. During EB-PVD deposition, ceramic columns develop through competitive growth, and differences introduced at the bond coat surface can consequently affect the subsequent evolution of the columnar architecture. Changes in column geometry and intercolumnar spacing may therefore persist through the coating thickness and become particularly apparent in the upper regions of the coating [18]. From a mechanical perspective, such variations are important because the local response of a columnar coating depends not only on the intrinsic properties of the ceramic phase but also on the arrangement and mechanical interaction of neighbouring columns and the volume fraction of intercolumnar gaps [19].

Despite numerous studies that addressed the influence of bond coat roughness on the nucleation, morphology, and crystallographic texture of EB-PVD coatings, the relationship between bond coat surface condition, the resulting columnar architecture, and the local mechanical response through the coating thickness remains insufficiently established. In particular, it is not clear whether changes in column morphology induced by bond coat roughness are accompanied by corresponding changes in hardness throughout the ceramic layer, or whether the effect is limited to specific regions of the evolving columnar structure.

Therefore, the objective of the present study was to systematically investigate the effect of bond coat surface roughness on the surface morphology, phase composition, columnar architecture, and mechanical response of EB-PVD 7 wt.% yttria-stabilised zirconia (7YSZ) thermal barrier coatings. Three distinct bond coat surface conditions were investigated, ranging from the as-coated state to ground and polished surfaces, while the EB-PVD deposition parameters were kept constant. The resulting coatings were characterised in terms of surface roughness, phase composition, and cross-sectional microstructure, including quantitative evaluation of column geometry, intercolumnar spacing, column inclination, and porosity. Their mechanical response was further evaluated by scratch testing and Vickers microindentation at selected positions through the coating thickness, from the vicinity of the bond coat interface to the column tips. The study specifically addresses whether the microstructural modifications induced by bond coat roughness are accompanied by corresponding changes in the local mechanical response and to what extent these effects can be distinguished from the intrinsic through-thickness mechanical gradient characteristic of EB-PVD columnar coatings.

## 2. Materials and Methods

### 2.1. Materials and Coating Deposition

Inconel 718 nickel-based superalloy was selected as the substrate material. Cylindrical samples with a diameter of 25.4 mm and a length of 10 mm were used. Before bond coat deposition, the substrates were grit-blasted with corundum at an impingement angle of 45° and a stand-off distance of 100 mm, followed by degreasing with isopropanol.

A vapour-phase aluminide (VPA) bond coat was subsequently deposited using a high-temperature, low-activity (HTLA) above-the-pack aluminisation process. The substrates were suspended on a grid above a powder mixture containing a Cr–Al alloy as the aluminium donor and NH_4_Cl as the halide activator. The aluminisation process was conducted in a semi-sealed retort under a high-purity argon atmosphere at a maximum temperature of 1050 °C for 5 h. The process promoted the outward diffusion of nickel and resulted in the formation of a homogeneous, predominantly single-phase β-NiAl diffusion coating with an average thickness of approximately 60 μm.

Following aluminisation, the bond coat surfaces were subjected to different surface treatments to obtain distinct roughness levels prior to ceramic top-coat deposition. One specimen was kept in as-coated condition, the other specimen was ground using 320-grit silicon carbide abrasive paper, while the surface of the third specimen was polished using a diamond suspension with a particle size of 3 μm. Samples were carefully cleaned after surface preparation to remove residual contaminants while preserving the resulting surface topography. To minimise differences in the thermal history of the bond coats resulting from the different surface preparation procedures, all samples were subjected to an identical heat treatment at 1050 °C for 30 min prior to EB-PVD deposition.

Commercial 7 wt.% yttria-stabilised zirconia (7YSZ) ingots (Phoenix Coating Resources Inc., currently Saint-Gobain Coating Solutions, Avignon, France), with diameters ranging from 62.5 to 62.9 mm, were used as the ceramic evaporation source. The 7YSZ composition was selected due to its established use in thermal barrier coating systems and its favourable combination of thermal stability, strain tolerance, and mechanical performance. Rapid vapour condensation of 7YSZ under non-equilibrium EB-PVD conditions promotes the formation of a non-transformable metastable tetragonal t′ phase, which provides improved phase stability compared to undoped or partially stabilised zirconia and limits detrimental transformation during thermal cycling.

The ceramic top coat was deposited in a single EB-PVD batch using a Smart Coater system (ALD Vacuum Technologies GmbH, Hanau, Germany). The specimen holder was positioned centrally above the evaporation pool in the middle-rake configuration. The holder remained stationary during deposition in order to maintain consistent deposition conditions for all specimens. The main deposition parameters were an electron-beam emission current of 3.02 A, a chamber pressure of 0.00925 mbar, a substrate temperature of 985.8 °C, a feed rate of 1.5 mm/min, and a deposition time of 900 s. The resulting 7YSZ top coats had an average thickness of approximately 165 μm.

### 2.2. Experimental Characterisation

The surface roughness of the bond coats prior to EB-PVD deposition was characterised using a stylus profilometer (Wave System, Hommelwerke, Villingen-Schwenningen, Germany). Measurements were taken at a constant stylus traverse speed of 0.5 mm/s over a measurement length of 15 mm. Ten measurements were taken for each sample, and the reported roughness parameters represent the arithmetic mean of the individual measurements. The surface roughness of the 7YSZ top coats was characterised using the same measurement system and measurement procedure, enabling a direct comparison of the surface topography before and after ceramic deposition.

The phase composition of the deposited 7YSZ top coats was analysed by X-ray diffraction (XRD). Diffraction patterns were collected over the 2θ range from 20° to 95° using the same measurement conditions for all coating variants. The cross-sectional microstructure of the 7YSZ coatings was characterised using a scanning electron microscope (SEM, Phenom XL, Thermo Fisher Scientific, Waltham, MA, USA).

Quantitative analysis of columnar architecture and porosity was performed using digital image processing of SEM cross-sectional micrographs in Fiji (ImageJ version 1.54p) software. Prior to analysis, the spatial scale of each micrograph was calibrated using the corresponding SEM scale bar. For each coating variant, ten representative areas were analysed to account for local microstructural variability, and the resulting values were averaged. The total porosity was determined by image segmentation. The grayscale threshold was adjusted for each micrograph to distinguish the ceramic matrix from pores and intercolumnar gaps. The total porosity was then calculated as the area fraction of the segmented void regions. Coating thickness, column width, and intercolumnar spacing were determined using the linear-intercept method at approximately 75% of the coating thickness, measured from the bond coat/top coat interface. The column inclination angle was determined using the angle measurement tool in Fiji by measuring the deviation of the column growth axis from the reference line corresponding to the bond coat surface.

Scratch resistance was evaluated using a Revetest system (CSM Instruments, Needham, MA, USA) equipped with a Rockwell-type diamond indenter with a nominal tip radius of 200 μm. A progressive normal load of 1 to 200 N was applied at a loading rate of 39.8 N/min on a scratch length of 10 mm. The scratching speed was maintained at 2 mm/min. Following testing, the scratch tracks were examined to identify evidence of coating cracking, delamination, spallation, or other forms of mechanical damage. The penetration depth of the indenter was also analysed as a function of the applied load.

The local mechanical response of the 7YSZ coatings was evaluated on polished cross-sections using a Micro-Combi Tester (CSM Instruments, Needham, MA, USA) equipped with a Vickers diamond indenter. A maximum indentation load of 500 mN was applied using a loading time of 30 s and an unloading time of 30 s, corresponding to an approximately constant loading and unloading rate of 16.7 mN/s. A dwell time of 10 s was maintained at the maximum load prior to unloading. Under these conditions, the maximum indentation depth was approximately 1.5–2.0 μm, corresponding to approximately 1–1.5% of the average coating thickness (~165 μm). This indentation depth-to-coating-thickness ratio was selected to minimise the influence of the underlying bond coat and substrate on the measured hardness response.

**Figure 1:**
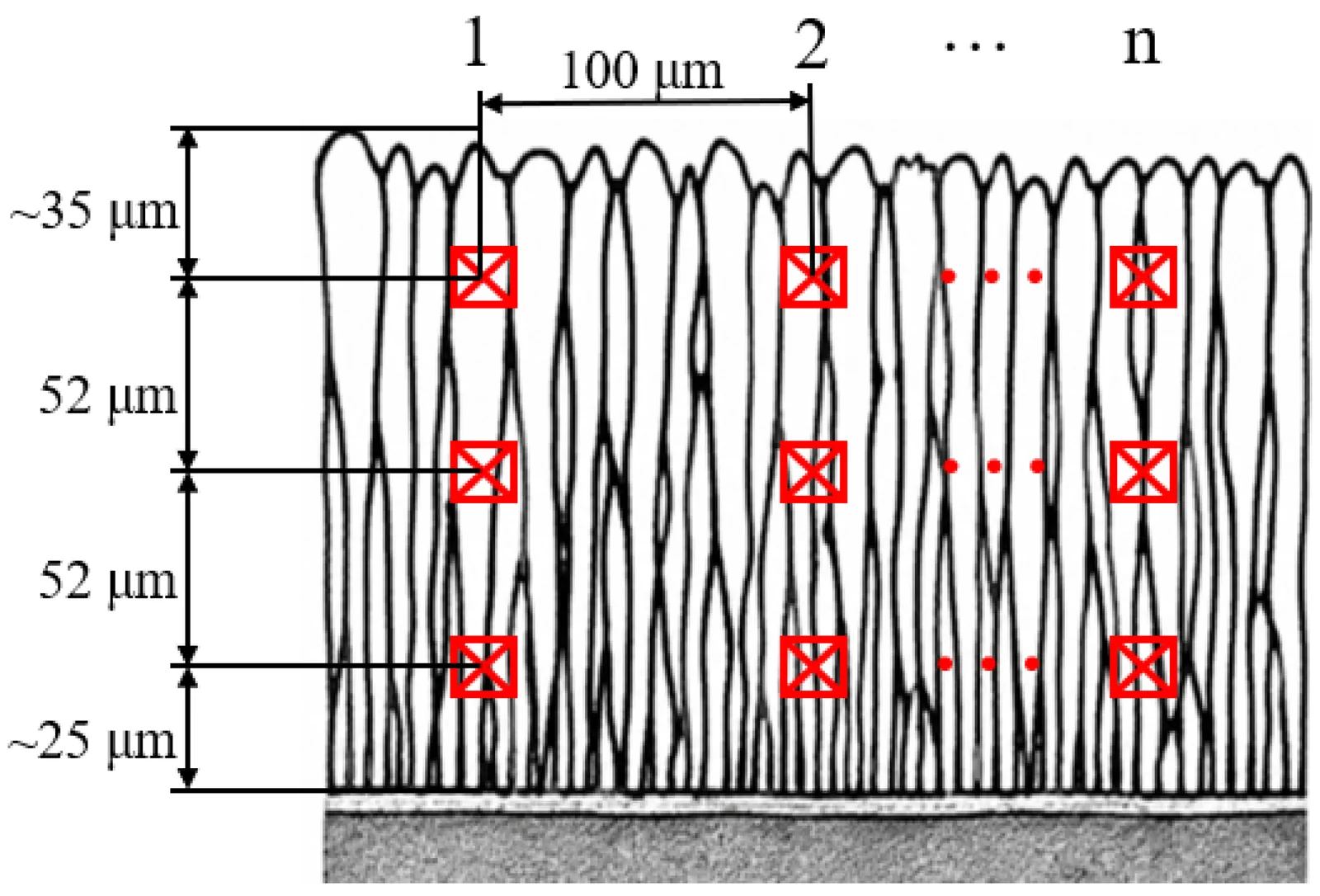
Schematic representation of the TBC cross-section. The red markers indicate the locations selected for indentation measurements.

Indentation measurements were performed in three characteristic regions through the thickness of the coating: near the interface between the bond coat and the ceramic top coat, in the central region of the ceramic coating, and close to the column tips. The centres of the indents were placed approximately 25 μm from the bond coat and 35 μm from the column tips, respectively (Figure 1). These distances were selected to minimise the influence of neighbouring interfaces while maintaining a representative assessment of the local mechanical response of the coating microstructure.

For each coating variant, twenty independent measurement series were performed. Each series consisted of three indents located in the regions described above, resulting in a total of sixty indents per specimen and twenty measurements for each investigated region. The spacing between neighbouring measurement series was maintained at 100 μm in order to eliminate interactions between adjacent plastic deformation zones and possible crack fields.

The diagonals of the Vickers imprints were measured optically using the instrument software. To minimise operator-dependent uncertainty, each indentation imprint was measured ten times, and the corresponding hardness values were averaged to obtain a single representative Vickers hardness value. Indents exhibiting chipping, spallation of the coating, or any other defects affecting the precision of diagonal measurements were excluded from further analysis. The indentation imprints and their immediate surroundings were additionally examined optically for evidence of microcracking or intercolumnar separation. No systematic micro-lateral cracking or intercolumnar delamination was observed around the valid indents used for hardness evaluation. No fewer than 15 valid measurements were retained for statistical evaluation in each investigated region.

### 2.3. Statistical Analysis

The normality of the data distribution was assessed using the Shapiro–Wilk test. As normality could not be confirmed for all datasets, statistical comparisons were performed using the nonparametric Kruskal–Wallis test followed by Dunn’s post hoc test with Bonferroni correction. Differences were considered statistically significant at *p* < 0.05.

## 3. Results and Discussion

### 3.1. Influence of Bond Coat Surface Condition on the Roughness of the Ceramic Top Coat

The surface roughness of the bond coat was found to have a pronounced effect on the surface topography of the EB-PVD 7YSZ top coat. The roughness parameters measured for the bond coats and the corresponding ceramic top coats are summarised in Table 1. A systematic decrease in the arithmetic mean roughness of the ceramic top coat was observed with decreasing bond coat roughness. For the as-coated bond coat, characterised by R_a_ = 0.893 ± 0.027 μm, the resulting 7YSZ top coat exhibited R_a_ = 0.826 ± 0.022 μm. After grinding the bond coat, its roughness decreased to R_a_ = 0.492 ± 0.048 μm, while the corresponding top coat exhibited R_a_ = 0.580 ± 0.041 μm. Further polishing reduced the roughness of the bond coat to R_a_ = 0.019 ± 0.001 μm and resulted in a further decrease in the roughness of the ceramic top coat to R_a_ = 0.398 ± 0.011 μm.

The reduction in top coat roughness with decreasing bond coat roughness is also reflected in the other profile parameters listed in Table 1. In particular, R_z_ decreased from 7.075 ± 0.744 μm for Top Coat I to 5.225 ± 0.561 μm for Top Coat II and 4.267 ± 0.371 μm for Top Coat III. A similar trend was observed for R_max_, which decreased from 10.116 ± 2.537 μm to 7.069 ± 1.334 μm and 6.655 ± 1.063 μm, respectively. These results demonstrate that the surface condition of the bond coat is transferred, at least partially, to the surface topography of the subsequently deposited EB-PVD ceramic layer.

**Figure 2:**
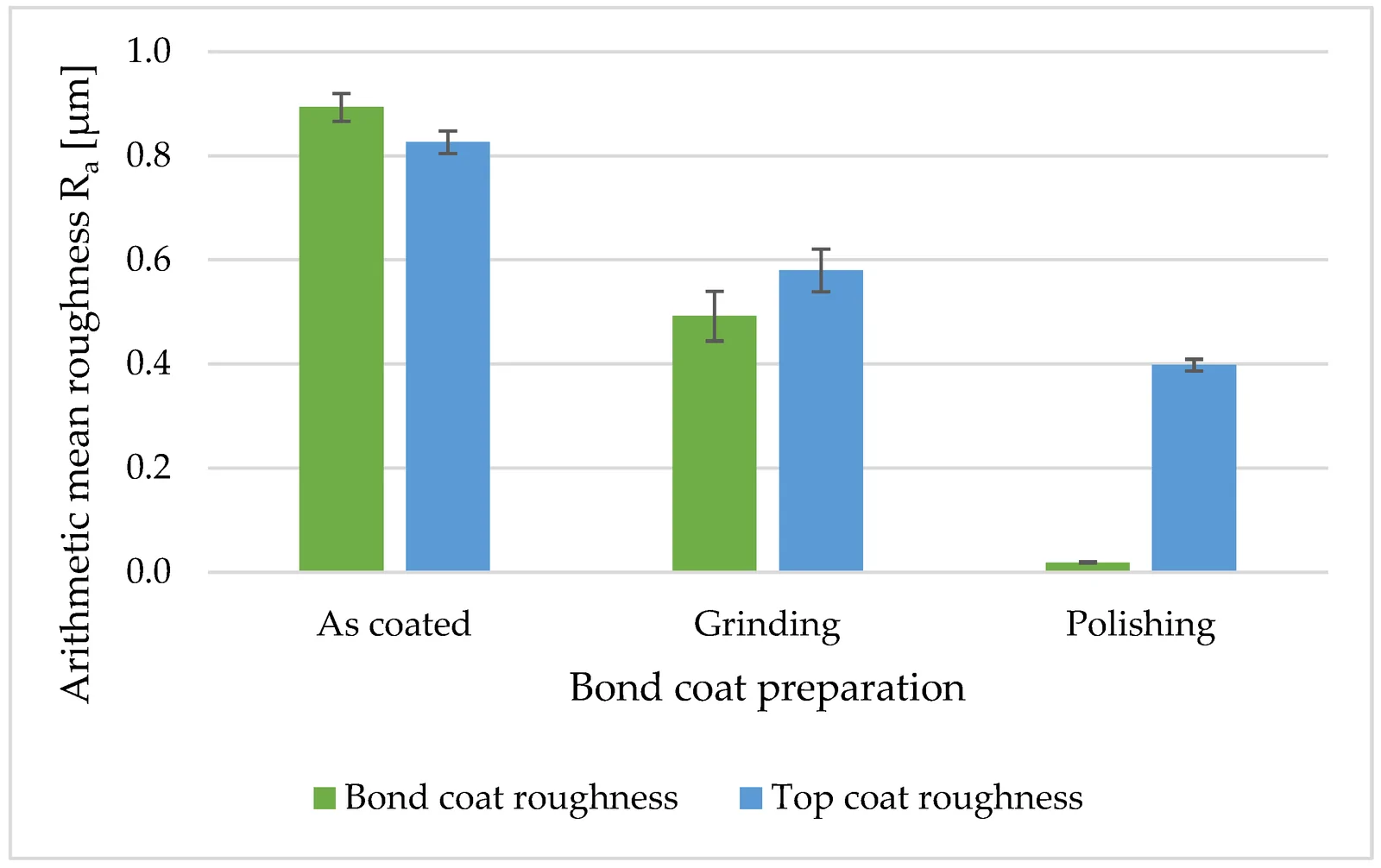
Effect of bond coat surface condition on the roughness of the EB-PVD 7YSZ top coat.

The relationship between the bond coat and top coat roughness is further illustrated in Figure 2. The reduction in bond coat roughness from the as-coated condition to the ground condition resulted in a pronounced decrease in the roughness of the ceramic layer. However, further reduction in the bond coat roughness by polishing produced a comparatively smaller decrease in the R_a_ value of the 7YSZ top coat. This indicates that the relationship between the substrate and top coat roughness is not linear over the entire investigated range. Once the bond coat surface becomes sufficiently smooth, the surface topography of the ceramic coating is increasingly governed by the morphology of the EB-PVD column tips rather than by the initial bond coat profile.

**Figure 3:**
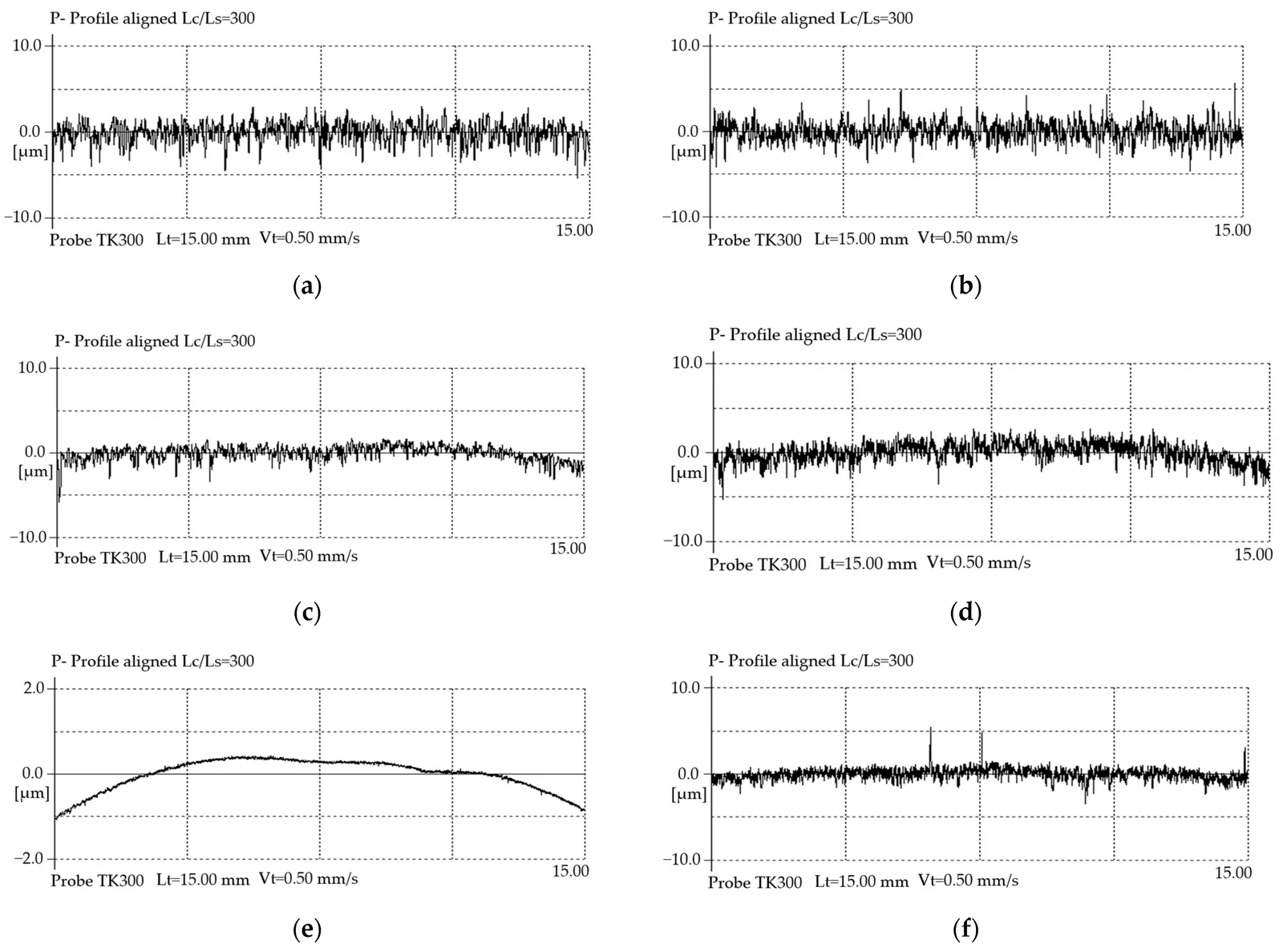
Representative surface roughness profiles measured using a contact profilometer for (**a**) Bond Coat I in the as-coated condition (R_a_ = 0.893 μm); (**b**) corresponding Top Coat I (R_a_ = 0.826 μm); (**c**) Bond Coat II after grinding (R_a_ = 0.492 μm); (**d**) corresponding Top Coat II (R_a_ = 0.580 μm); (**e**) Bond Coat III after polishing (R_a_ = 0.019 μm); and (**f**) corresponding Top Coat III (R_a_ = 0.398 μm).

The profilometric profiles presented in Figure 3 provide direct visual evidence of this relationship. For the as-coated bond coat, pronounced surface irregularities were retained after deposition of the ceramic layer (Figure 3a,b). Grinding substantially reduced the amplitude of the bond coat profile and resulted in a corresponding reduction in the top coat’s surface roughness (Figure 3c,d). On the contrary, polishing reduced bond coat roughness to nearly R_a_ = 0.02 μm, whereas the corresponding ceramic coating still exhibited R_a_ = 0.398 μm (Figure 3e,f). Thus, the surface roughness of the EB-PVD top coat cannot be attributed solely to the initial roughness of the bond coat. Instead, it represents the combined effect of substrate topography and the morphology of the growing columnar structure. The observed relationship is in agreement with previously reported results [16].

### 3.2. Phase Composition and Crystallographic Characteristics of the 7YSZ Top Coats

**Figure 4:**
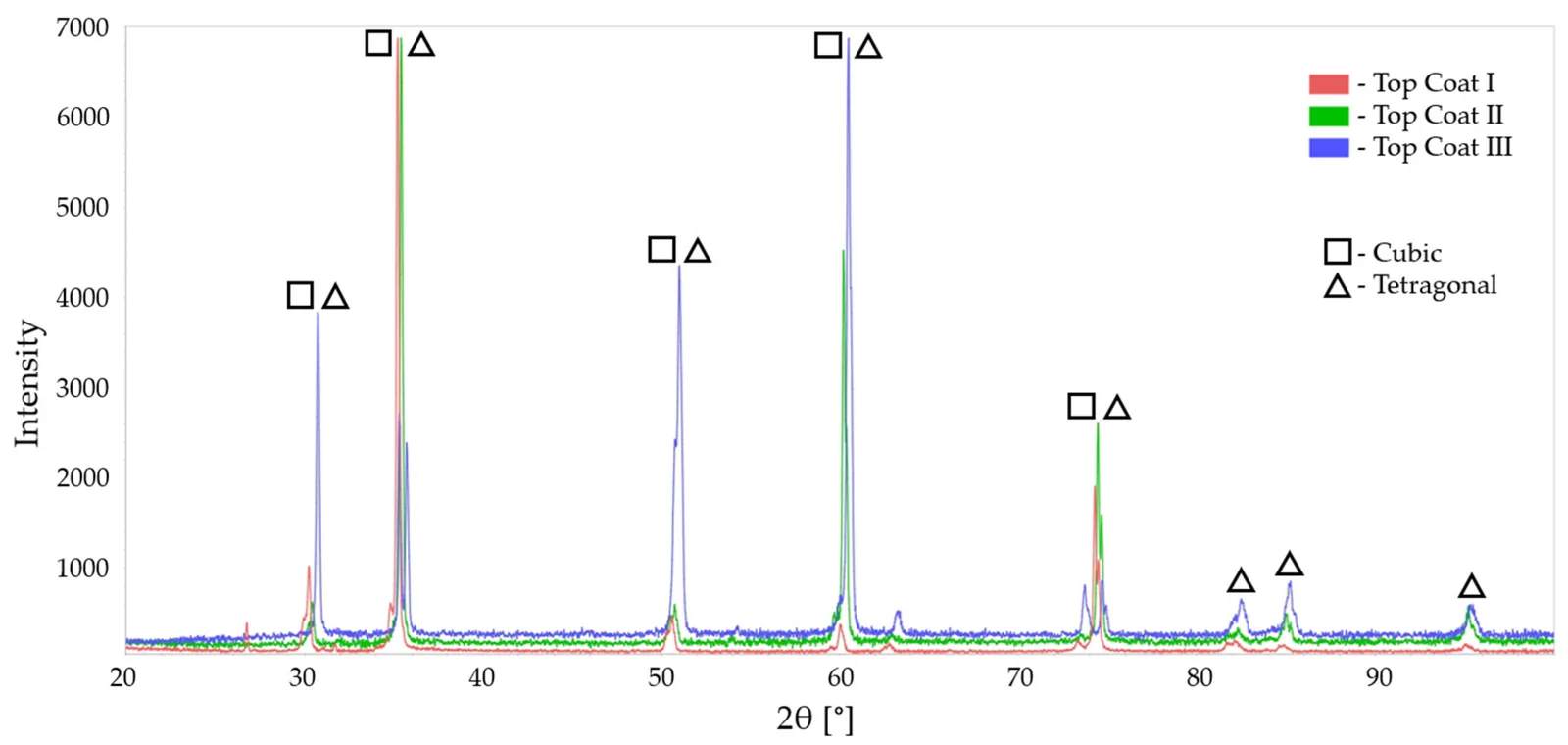
X-ray diffraction patterns of the 7YSZ top coats deposited on bond coats with different surface conditions. The diffraction peaks were indexed according to the tetragonal and cubic YSZ reference patterns (ICDD PDF No. 48-0224 and No. 30-1468).

X-ray diffraction (XRD) analysis was performed to verify the phase composition of the 7YSZ top coats and to assess whether modification of the bond coat surface roughness resulted in detectable changes in their crystallographic characteristics. The diffraction patterns obtained for all investigated coatings are presented in Figure 4.

All coatings exhibited diffraction peaks at comparable 2θ positions, indicating a similar crystal structure and confirming the formation of the intended yttria-stabilised zirconia top coat regardless of the bond coat surface treatment. The diffraction peaks were indexed according to the tetragonal YSZ reference pattern (ICDD PDF No. 48-0224). The principal reflections observed at approximately 30.2°, 35.0°, 50.2°, 60.0°, 62.7°, 74.2°, 81.7°, and 84.8° correspond to the (101), (110), (112), (211), (202), (220), (213), and (310) planes, respectively. Due to the substantial overlap between the reflections of tetragonal and cubic YSZ, the presence of a minor cubic contribution cannot be unambiguously resolved from the present θ–2θ diffraction patterns. No additional diffraction peaks attributable to undesired secondary phases were identified within the investigated angular range. The diffraction patterns were consistent with the characteristic metastable t′-YSZ structure expected for as-deposited EB-PVD 7YSZ coatings [20,21,22,23,24]. Importantly, the similarity of the diffraction patterns confirms that the different bond coat surface treatments did not prevent the formation of the intended 7YSZ ceramic top coat.

Although the positions of the diffraction peaks remained essentially unchanged, differences in their relative intensities were observed between the investigated coatings. These variations may be associated with differences in crystallographic texture or preferred orientation resulting from changes in the initial surface condition of the bond coat. However, the present X-ray diffraction measurements do not provide sufficient evidence to quantify these effects or to establish a direct relationship between bond coat roughness and the degree of crystallographic texture. Therefore, the observed changes in the peak intensity are considered indicative of differences in crystallographic orientation rather than evidence of a change in phase composition.

### 3.3. Effect of Bond Coat Roughness on the Columnar Microstructure of the 7YSZ Top Coat

**Figure 5:**
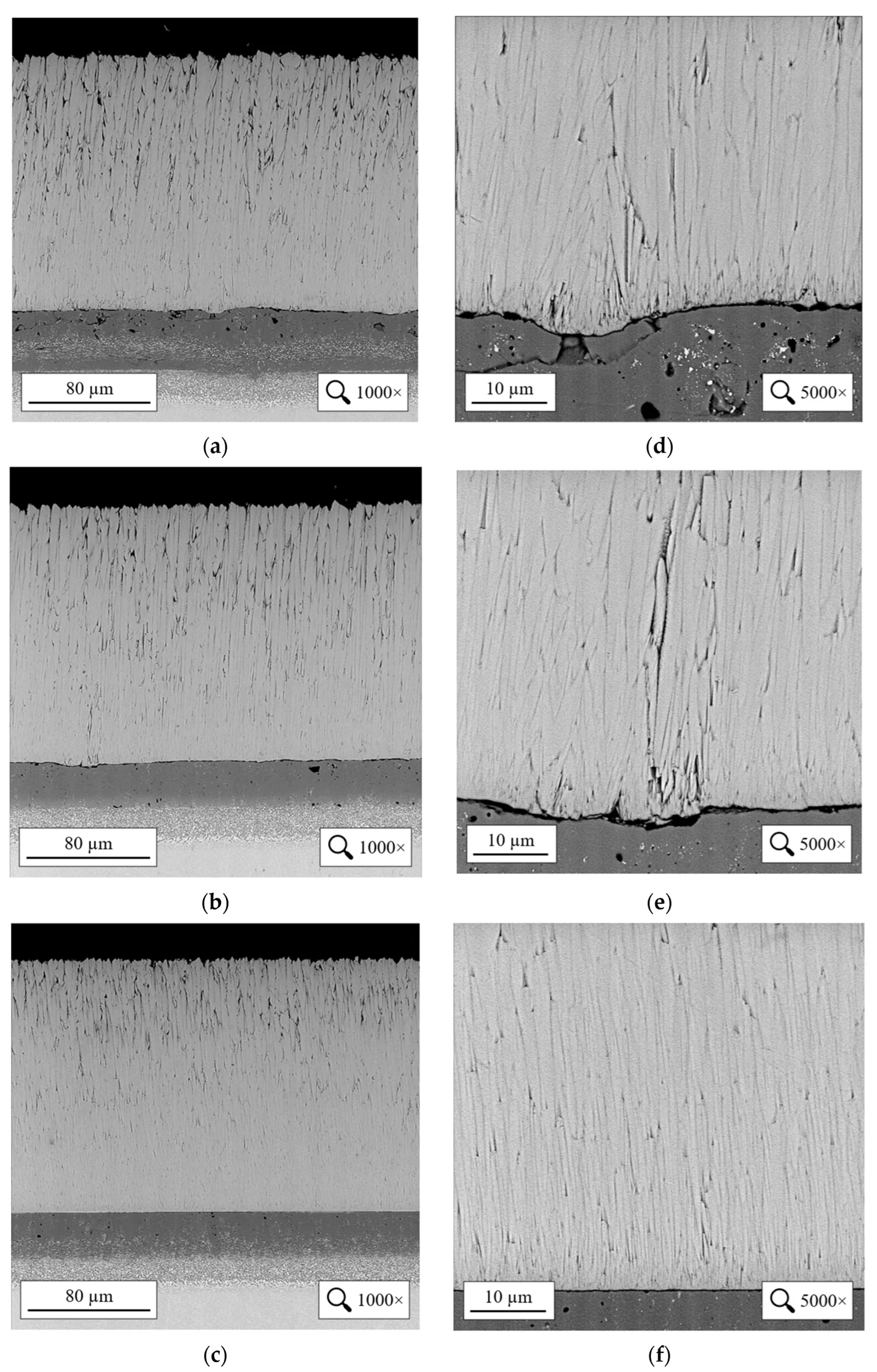
Cross-sectional SEM micrographs of the 7YSZ top coats deposited on: (**a**) sample I, as-coated bond coat; (**b**) sample II, ground bond coat; and (**c**) sample III, polished bond coat. Figures (**d**–**f**) show higher-magnification views of the corresponding microstructures.

The cross-sectional microstructures of the EB-PVD 7YSZ top coats deposited on bond coats with different surface conditions are presented in Figure 5. A clear relationship was observed between the initial bond coat topography and the resulting columnar architecture. With decreasing bond coat surface roughness, the columnar structure became progressively more uniform, with a more consistent growth direction and reduced variation in column width and intercolumnar spacing.

For the coating deposited on the as-coated bond coat, the columns exhibited greater variation in morphology and orientation, accompanied by relatively wide and irregular intercolumnar gaps (Figure 5a,d). In contrast, grinding of the bond coat surface resulted in a more homogeneous columnar structure (Figure 5b,e). The most pronounced change was observed for sample III, deposited on the polished bond coat. In this case, the columns were more closely spaced and exhibited a more uniform orientation throughout the coating thickness (Figure 5c,f).

The quantitative analysis of the columnar architecture supports the qualitative observations from the SEM images (Table 2). A progressive reduction in column width was observed with a decrease in bond coat roughness, from 4.91 ± 1.89 μm for sample I to 4.33 ± 1.54 μm for sample II and 3.90 ± 1.39 μm for sample III. At the same time, the average intercolumnar spacing decreased from 0.83 ± 0.32 μm to 0.79 ± 0.28 μm and 0.73 ± 0.22 μm, respectively. Thus, surface smoothing of the bond coat promoted the formation of a finer and more closely packed columnar architecture.

A similar trend was observed for the overall porosity of the coatings. The measured porosity decreased from 5.05 ± 0.50% for sample I to 4.84 ± 0.57% for sample II and 3.86 ± 0.76% for sample III. This reduction is consistent with the observed decrease in intercolumnar spacing and indicates that the initial bond coat topography affects not only the geometrical arrangement of individual columns but also the overall open volume of the ceramic layer.

The orientation of the columns was also affected by the bond coat’s surface condition. The average column inclination angle increased from 83.28 ± 0.95° for sample I to 85.88 ± 0.85° for sample II and 87.82 ± 1.21° for sample III. Therefore, smoothing of the bond coat promoted more perpendicular and uniform columnar growth relative to the substrate surface.

**Figure 6:**
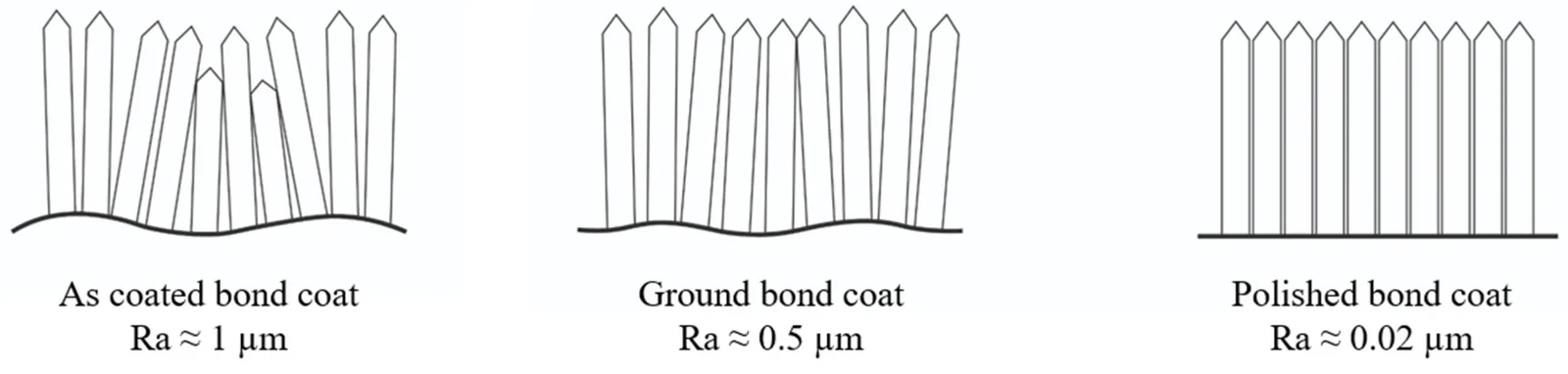
Schematic representation of the effect of bond coat surface roughness on the columnar architecture of the EB-PVD 7YSZ top coat.

The observed changes can be related to the role of the initial substrate topography during the nucleation and early growth stages of EB-PVD deposition. In EB-PVD, the local surface geometry influences the orientation and shadowing conditions of the vapour flux during the initial stages of coating formation. Pronounced surface asperities may result in locally different nucleation conditions and preferential growth directions. As the coating develops, these differences can be transferred into the growing columnar architecture, leading to variations in column orientation, width, and intercolumnar spacing. By contrast, the smoother bond coat surface provides a more uniform initial growth interface. This favours the formation of columns with a more consistent orientation and reduces the geometrical variability introduced during the early stages of deposition. The SEM observations therefore demonstrate that bond coat surface roughness is not merely transferred to the surface topography of the ceramic coating, as discussed in Section 3.1, but also influences the internal architecture of the EB-PVD 7YSZ layer (Figure 6).

### 3.4. Effect of Bond Coat Roughness on the Scratch-Test Response of EB-PVD TBCs

Scratch testing was performed to assess the mechanical response and damage tolerance of the EB-PVD 7YSZ top coats deposited on bond coats with different surface conditions. No visible delamination, spallation, or extensive cracking of the ceramic top coat was observed for any of the investigated coating variants throughout the applied load range. Therefore, within the experimental conditions used in the present study, none of the coatings exhibited a pronounced loss of coating integrity. The absence of a distinct catastrophic damage event indicates a relatively high resistance of the investigated TBC systems to the imposed scratching load.

**Figure 7:**
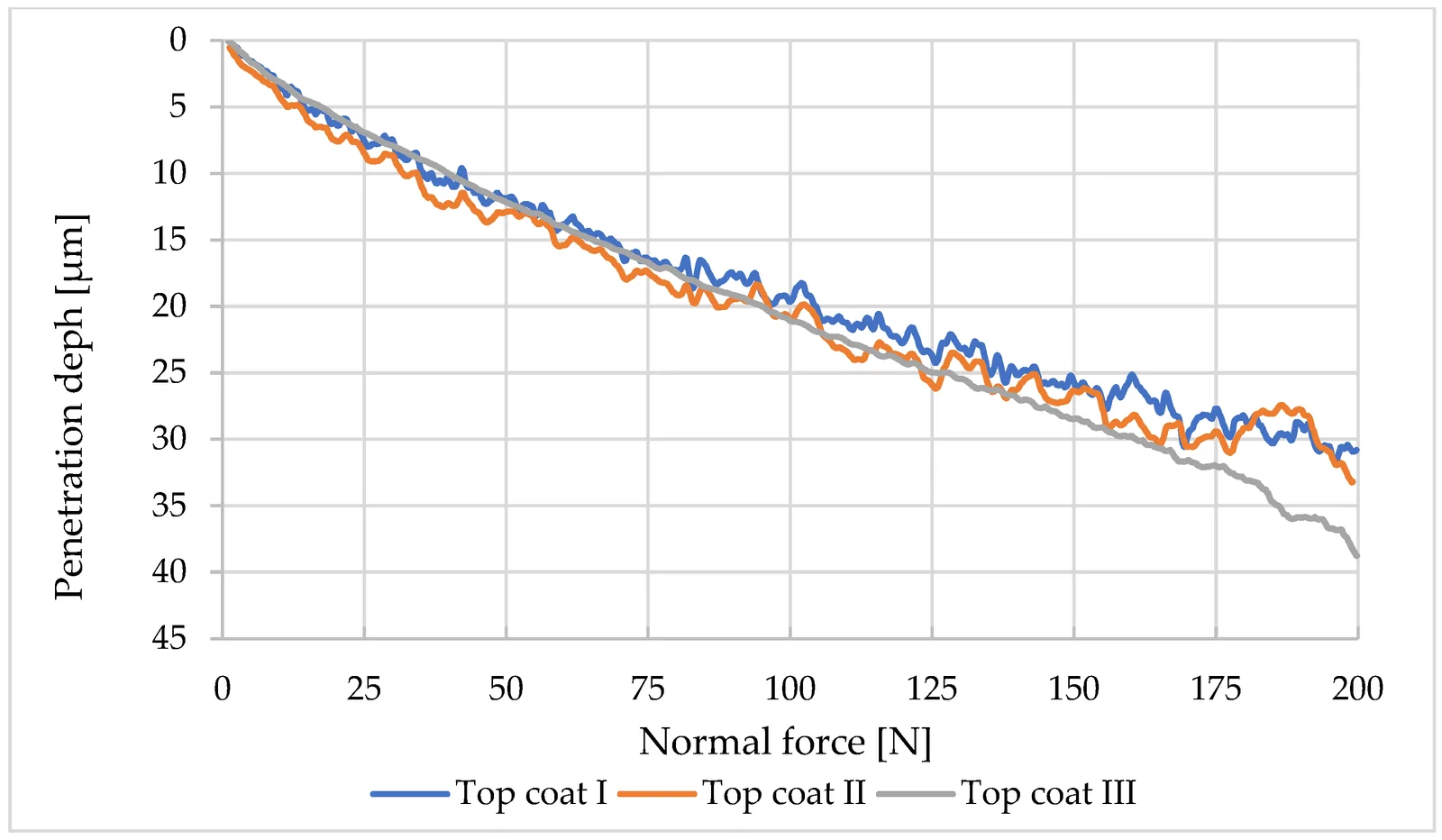
Representative scratch-test response of the EB-PVD 7YSZ top coats deposited on bond coats with different surface conditions.

The variation in indenter penetration depth during the scratch test is presented in Figure 7. At the maximum normal load of 200 N, the penetration depth was approximately 31 μm for the coating deposited on the as-coated bond coat. An increase in penetration depth was observed for coatings deposited on progressively smoother bond coat surfaces, reaching approximately 34 μm for the ground condition and 39 μm for the polished condition. The observed differences in penetration depth indicate that the local mechanical response of the ceramic top coat was affected by the bond coat surface condition. This behaviour is consistent with the microstructural observations presented in Section 3.3, which demonstrated systematic changes in column width, intercolumnar spacing, column inclination, and overall porosity with decreasing bond coat roughness. These structural differences can modify the local compliance and load-bearing response of the coating during scratching.

An important limitation of the scratch test in the present study is the relatively shallow penetration of the indenter compared to the total coating thickness, which was approximately 170 μm in the samples subjected to the scratch test. Consequently, the scratch response was dominated by the upper and near-surface portion of the columnar architecture, rather than representing the mechanical response of the coating uniformly throughout its entire thickness. This is particularly relevant because the SEM analysis demonstrated that the morphology of the columnar structure changes systematically as a function of bond coat roughness. Therefore, the scratch-test results provide complementary evidence that the bond coat surface condition influences the mechanical response of the resulting EB-PVD coating. However, because the scratch test probes a relatively shallow and mechanically heterogeneous region of the columnar coating, it does not allow the through-thickness hardness distribution to be determined. For this reason, Vickers indentation measurements were employed to quantify the hardness distribution throughout the coating thickness.

### 3.5. Through-Thickness Hardness Distribution and the Effect of Bond Coat Roughness

**Figure 8:**
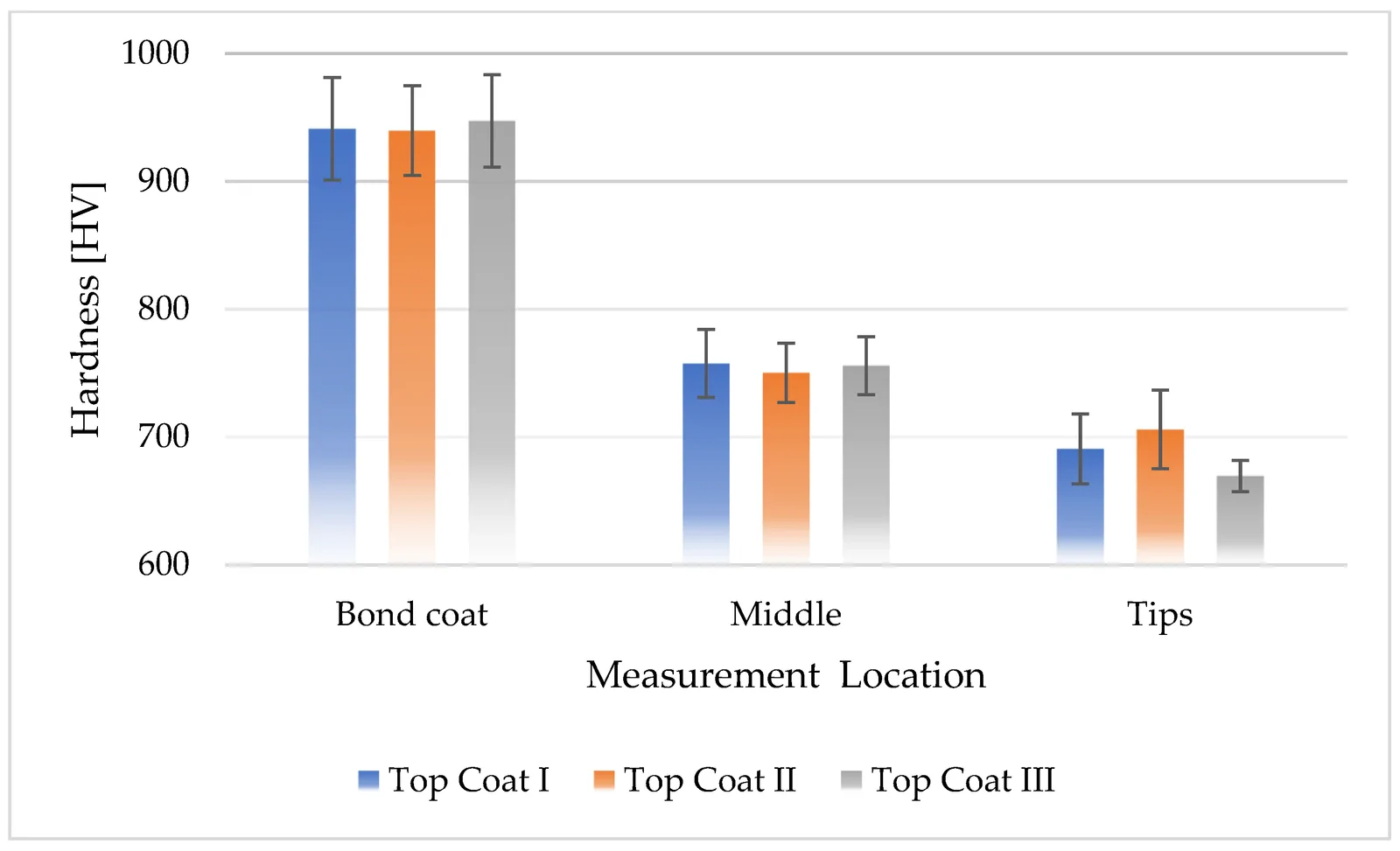
Comparison of average Vickers hardness values measured in the three investigated regions of the coatings. The error bars represent the standard deviations.

Indentation tests were performed in three characteristic regions of the ceramic top coat, namely in the vicinity of the bond coat, in the central part of the coating, and near the column tips. The average Vickers hardness values of the 7YSZ EB-PVD coatings obtained in the three investigated regions are given in Table 3 and presented in Figure 8. Significant differences in hardness were observed between the investigated regions of the top coat for all coating variants. The hardness decreased progressively from the bond coat interface towards the column tips. The highest hardness values were consistently measured in the vicinity of the bond coat; intermediate values were recorded in the central region of the coating, while the lowest hardness values were observed near the column tips. The Kruskal–Wallis test confirmed statistically significant differences between the investigated regions for coating I (H = 42.21, *p* = 6.83 × 10^−10^), coating II (H = 35.80, *p* = 1.68 × 10^−8^), and coating III (H = 40.85, *p* = 1.35 × 10^−9^). Subsequent Dunn–Bonferroni post-hoc analysis revealed statistically significant differences between all pairwise comparisons (bond coat region vs. middle region, bond coat region vs. tips region, and middle region vs. tips region) for each coating variant (*p* < 0.01). The mean hardness values ranged from approximately 940 to 947 HV in the vicinity of the bond coat to 750–758 HV in the central region and 669 to 706 HV near the column tips.

The presence of a statistically significant hardness gradient through the coating thickness indicates substantial variations in the local mechanical response associated with the characteristic EB-PVD columnar architecture. In the vicinity of the bond coat, the high density of columns and the limited volume fraction of the intercolumnar gaps provide greater resistance to plastic deformation under indentation loading. With increasing distance from the bond coat interface, the influence of intercolumnar porosity becomes progressively more pronounced, reducing the effective load-bearing area and facilitating local deformation. Consequently, the lowest hardness values were observed in the vicinity of the column tips, where the microstructure is comparatively more open. Importantly, the same hardness profile was observed for all investigated coating variants despite noticeable differences in column morphology. This finding suggests that the through-thickness hardness gradient is primarily an intrinsic feature of EB-PVD coatings and originates from the evolution of the columnar structure during growth rather than from variations in bond coat preparation. These observations are consistent with those presented in reference [18].

**Figure 9:**
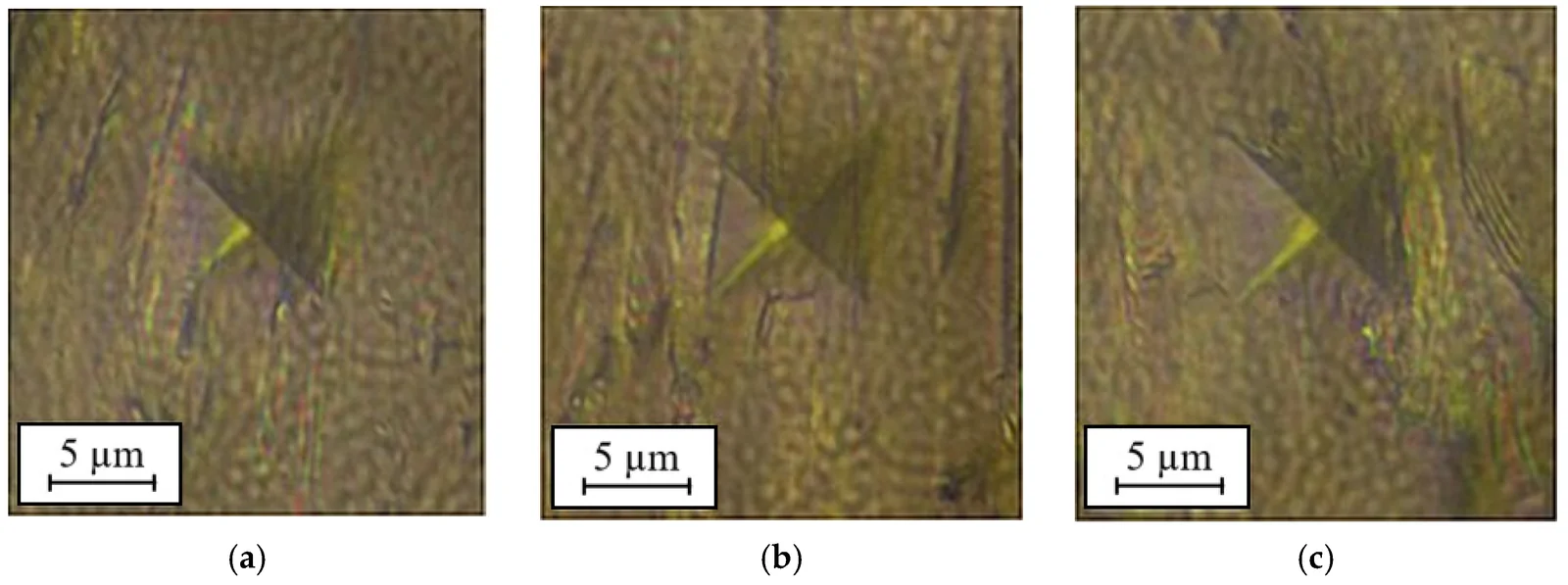
Representative Vickers imprints obtained: (**a**) in the vicinity of the bond coat, (**b**) in the middle of the coating, and (**c**) near the column tips.

Representative Vickers imprints obtained in different regions of the coating are presented in Figure 9. The indentation size progressively increased from the bond coat towards the column tips. The smallest imprints were consistently observed in the lower region of the coating, whereas larger impressions were formed in the central and upper regions. This observation agrees well with the measured hardness distribution and provides direct visual confirmation of the through-thickness mechanical gradient.

The influence of bond coat surface roughness on local hardness was further assessed by comparing the hardness values obtained for coatings deposited on bond coats prepared using different surface treatments. Statistical analysis did not reveal significant differences between coating variants in the vicinity of the bond coat (Kruskal–Wallis, *p* = 0.75) or in the central region of the coating (*p* = 0.69). By contrast, statistically significant differences were detected near the column tips (*p* < 0.001). Dunn–Bonferroni post hoc analysis demonstrated that coating III exhibited significantly lower hardness values than coatings I and II, while no significant differences were found between coatings I and II.

The absence of statistically significant differences in the lower and central regions of the coating indicates that the investigated bond coat preparation procedures did not substantially affect the mechanical response of the majority of the ceramic layer. However, the significant differences observed near the column tips suggest that the bond coat surface roughness influences the final stages of column growth during EB-PVD deposition. SEM observations support this interpretation, revealing distinct differences in column morphology in the upper part of the coating. In particular, coatings deposited on polished bond coats exhibited noticeably finer column tips compared to coatings grown on rougher bond coats. These microstructural differences are likely responsible for the observed variations in local hardness and demonstrate that the effect of bond coat roughness is most pronounced in the upper region of the coating.

From a thermomechanical perspective, the lower hardness measured near the column tips may indicate a locally more compliant response of the uppermost coating region. Together with the intercolumnar gaps characteristic of EB-PVD microstructures, this local compliance may facilitate accommodation of transient thermally induced strains and reduce stress concentrations during rapid temperature changes. Yang et al. [25] demonstrated that TBC damage evolution during thermal cycling depends on the stage of the cycle, with vertically oriented cracks developing predominantly during heating and interfacial damage becoming more significant during cooling. Therefore, the lower hardness observed near the column tips, particularly for sample III, may contribute to strain accommodation in the upper part of the coating. Nevertheless, hardness alone cannot be considered a direct measure of thermal-shock resistance or coating lifetime, which also depend on fracture resistance, residual stress state, interfacial integrity, and TGO evolution. Since thermal shock and thermal-cycling experiments were beyond the scope of the present study, this interpretation should be considered a hypothesis that requires dedicated experimental verification.

It should be noted that the present results describe the as-deposited condition. During prolonged high-temperature exposure, YSZ coatings may undergo sintering-induced densification, accompanied by a reduction in pore and intercolumnar gap dimensions and an increase in local stiffness and hardness [26,27]. The more compact architecture obtained for sample III, characterised by the smallest intercolumnar spacing and lowest total porosity, may favour the formation of sintered bridges between adjacent columns during thermal exposure. Such a microstructural evolution could modify both the hardness distribution and the strain-accommodation capability of the coating, particularly in the upper region. The verification of this hypothesis requires dedicated thermal-exposure and thermal-cycling experiments and is beyond the scope of the present study.

## 4. Conclusions

The present study demonstrated that the surface condition of the bond coat plays an important role in determining the surface topography and columnar architecture of EB-PVD 7YSZ thermal barrier coatings, while its effect on the local mechanical response is more limited. Based on the combined surface, phase, microstructural, and mechanical characterisation, the following conclusions can be drawn:

1. The surface condition of the bond coat was effectively transferred to the ceramic top coat. A progressive reduction in bond coat roughness resulted in a corresponding reduction in the surface roughness of the deposited 7YSZ layer. However, this relationship was not linear over the entire investigated roughness range. Further polishing of an already smooth bond coat produced only a limited additional reduction in top-coat roughness, indicating that the surface morphology of the EB-PVD column tips becomes an increasingly dominant contribution to the final coating topography.
2. Bond coat roughness substantially affected the architecture of the EB-PVD columnar microstructure. SEM observations and quantitative image analysis revealed more uniform column morphology, reduced intercolumnar spacing, increased column density, and a more consistent growth direction with decreasing bond coat roughness. The quantitative analysis also showed a systematic reduction in total porosity from the roughest bond coat condition to the smoothest. These results demonstrate that the initial surface topography of the bond coat influences the nucleation and subsequent competitive growth of the 7YSZ columns.
3. A pronounced through-thickness hardness gradient was identified in all EB-PVD coatings. Vickers microindentation revealed statistically significant differences between the regions near the bond coat, in the centre of the coating, and near the column tips. The highest hardness was consistently measured near the bond coat, whereas the lowest values were obtained near the column tips. This gradient was statistically significant for all three coating variants and indicates that the local mechanical response is strongly associated with the characteristic evolution of the columnar architecture through the coating thickness.
4. The influence of bond coat roughness on hardness was considerably weaker than its influence on microstructure. No statistically significant differences in hardness between the three coating variants were detected near the bond coat or in the central region of the ceramic layer. Statistically significant differences were observed only near the column tips, where the coating deposited on the smoothest bond coat exhibited a lower hardness than the coatings deposited on the rougher surfaces. Combined with the SEM observations, this indicates that bond coat roughness primarily affects the later stages of columnar growth and, consequently, the local mechanical response of the uppermost coating region.
5. The results indicate that bond coat roughness acts primarily as a microstructural control parameter rather than as a direct determinant of the hardness of the entire 7YSZ top coat. Although substantial changes in column morphology, intercolumnar spacing, porosity, and surface topography were produced by modifying the bond coat condition, the dominant through-thickness hardness gradient remained present for all coating variants. The mechanical response of the coating is therefore governed by the combined effect of the intrinsic EB-PVD columnar architecture and its local evolution with coating thickness, while the effect of bond coat roughness becomes most apparent in the vicinity of the column tips.

## Figures and Tables

**Table 1:** Surface roughness and profile parameters of the investigated bond coats and corresponding EB-PVD 7YSZ top coats.

Sample	Bond Coat Preparation	TBC Coating Layer	R_a_ [µm]	R_z_ [µm]	R_max_ [µm]	R [µm]
I	As coated	Bond Coat I	0.893 ± 0.027	6.599 ± 0.337	7.922 ± 1.111	3.737 ± 0.091
		Top Coat I	0.826 ± 0.022	7.075 ± 0.744	10.116 ± 2.537	3.516 ± 0.117
II	Grinding	Bond Coat II	0.492 ± 0.048	4.167 ± 0.523	4.686 ± 0.325	2.025 ± 0.157
		Top Coat II	0.580 ± 0.041	5.225 ± 0.561	7.069 ± 1.334	2.545 ± 0.178
III	Polishing	Bond Coat III	0.019 ± 0.001	0.162 ± 0.039	0.272 ± 0.149	0.056 ± 0.003
		Top Coat III	0.398 ± 0.011	4.267 ± 0.371	6.655 ± 1.063	1.733 ± 0.039

Note: R_a_—arithmetic mean roughness. R_z_—mean roughness depth. R_max_—maximum roughness depth. R—mean height of profile elements. Values are presented as mean ± standard deviation.

**Table 2:** Quantitative architectural parameters and porosity of EB-PVD 7YSZ coatings deposited on bond coats with different surface roughness.

Sample	Bond Coat Preparation	Coating Thickness [μm]	Column Width [μm]	Column Spacing [μm]	Column Inclination Angle [°]	Total Porosity [%]
I	As coated	162.4 ± 2.2	4.91 ± 1.89	0.83 ± 0.32	83.28 ± 0.95	5.05 ± 0.50
II	Grinding	164.7 ± 3.1	4.33 ± 1.54	0.79 ± 0.28	85.88 ± 0.85	4.84 ± 0.57
III	Polishing	164.4 ± 3.2	3.90 ± 1.39	0.73 ± 0.22	87.82 ± 1.21	3.86 ± 0.76

**Table 3:** Average Vickers hardness values measured at different through-thickness locations of the investigated 7YSZ top coats.

Measurement Location	Top Coat I Hardness [HV]	Top Coat II Hardness [HV]	Top Coat III Hardness [HV]
Near the Bond Coat	941 ± 40	940 ± 35	947 ± 36
In the Middle	758 ± 27	750 ± 23	756 ± 23
Close to the Tips	691 ± 27	706 ± 31	669 ± 12

## Data Availability

The original contributions presented in this study are included in the article. Further inquiries can be directed to the corresponding author.

## Abbreviations

The following abbreviations are used in this manuscript:

TBC	Thermal Barrier Coating
EB-PVD	Electron-Beam Physical Vapour Deposition
SEM	Scanning Electron Microscopy
HV	Hardness Vickers
